# Irrigation of *Suaeda salsa* with Saline Wastewater and Microalgae: Improving Saline–Alkali Soil and Revealing the Composition and Function of Rhizosphere Bacteria

**DOI:** 10.3390/microorganisms13071653

**Published:** 2025-07-12

**Authors:** Qiaoyun Yan, Yitong Zhang, Zhenting Xu, Wenying Qu, Junfeng Li, Wenhao Li, Chun Zhao, Hongbo Ling

**Affiliations:** 1 College of Water Conservancy and Architecture Engineering, Shihezi University, Shihezi 832000, China; 20222110020@stu.shzu.edu.cn (Q.Y.); 20221010007@stu.shzu.edu.cn (Y.Z.); 20221010026@stu.shzu.edu.cn (Z.X.); ljfshz@126.com (J.L.); lwh8510012@163.com (W.L.); 2 Key Laboratory of Modern Water-Saving Irrigation of Xinjiang Production and Construction Group, Shihezi University, Shihezi 832000, China; 3 Key Laboratory of Northwest Oasis Water-Saving Agriculture, Ministry of Agriculture and Rural Affairs, Shihezi 832000, China; 4 Key Laboratory of the Three Gorges Reservoir Region’s Eco-Environment, Ministry of Education, Chongqing University, Chongqing 400045, China; 5 Xinjiang Institute of Ecology and Geography, Chinese Academy of Sciences, Urumqi 830011, China; linghb@ms.xjb.ac.cn

**Keywords:** saline wastewater, microalgae, *Suaeda salsa*, saline-alkali land, bacterial community, carbon and nitrogen metabolism

## Abstract

Limited research has been conducted on the potential and mechanisms of irrigating *Suaeda salsa* with wastewater and microalgae to improve saline–alkali land. This study used three irrigation treatments (freshwater, saline wastewater, and saline wastewater with microalgae) to irrigate *S. salsa*, and microalgae promoted the growth of *S. salsa* and increased soil nutrient content, increasing available nitrogen (4.85%), available phosphorus (44.51%), and organic carbon (24.05%) while alleviating salt stress through reduced soil salinity (13.52%) and electrical conductivity (21.62%). These changes promoted eutrophic bacteria while inhibiting oligotrophic bacteria. Bacterial community composition exhibited significant variations, primarily driven by soil pH, total nitrogen, and organic carbon content. Notably, rhizosphere bacteria showed enhanced functional capabilities, with increased abundance of salt stress resistance and nitrogen metabolism-related genes compared to original soil, particularly under saline irrigation conditions. Furthermore, microalgae addition enriched nitrogen metabolism-related gene abundance. These findings revealed the potential role of key bacteria in enhancing plant growth and the soil environment and highlighted the potential of applying *S. salsa*, wastewater, and microalgae for the synergistic improvement of saline–alkali land.

## 1. Introduction

Land resources are essential for human survival. However, land salinization has become a severe issue in Xinjiang, China, significantly threatening these resources [1]. Salinization severely restricts crop growth, making the remediation of saline–alkali soils critical for ensuring food security and promoting sustainable ecosystem development [2]. Currently, the main measures for saline–alkali soil improvement include methods involving engineering, agronomic, and chemical means, among other measures. Although these techniques can partially improve soil quality, they are often costly and may cause secondary environmental pollution [3]. Consequently, developing sustainable strategies for saline–alkali soil amelioration has gained increasing research attention. For instance, recent studies have explored the use of halophytes [4] and microalgae-based biofertilizers [5] as sustainable solutions to improve saline–alkali soils.

As a halophytic pioneer species, *Suaeda salsa* demonstrates remarkable adaptability to saline–alkali soils and serves as valuable forage for livestock [6]. Studies have shown that planting *S. salsa* leads to notable improvements in soil properties, including reduced soil salinity, increased organic matter and available nitrogen content, thereby contributing significantly to the ecological restoration of microenvironments [7]. Microalgae, as photosynthetic microorganisms capable of converting light, water, carbon, and inorganic salts into biomass and nutrients, show great potential for improving saline–alkali soil [5]. Ma et al. [8] indicated that microalgae fertilizer has dual benefits for saline–alkali soil ecosystems: a significant restoration effect on saline–alkali land coupled with improving the photosynthetic capacity of quinoa, resulting in an increase in quinoa yield. The results of Shayesteh et al. [9] confirmed that microalgae influence soil biological processes and enhance the availability of soil nutrients. Moreover, the study highlighted the potential of heterotrophic nitrification in the soil amended with microalgae. These findings suggest that the application of microalgae could simultaneously improve saline–alkali soil quality and promote *S. salsa* biomass production. Furthermore, the synergistic combination of microalgae and *S. salsa* may provide even stronger improvements in saline–alkali soil remediation.

In Xinjiang, China, where water scarcity and severe soil salinization coexist, the integrated utilization of saline–alkali land and wastewater holds significant potential for sustainable agricultural development. Recent studies have demonstrated that wastewater containing appropriate nitrogen, phosphorus, and potassium can improve soil physicochemical characteristics and the composition of the microbial community, thereby effectively promoting plant growth and productivity [10]. However, the underlying mechanisms by which wastewater and microalgae irrigation promote the growth of *S. salsa* and soil amelioration remain unclear, thus limiting the widespread implementation of microalgae biofertilizers and wastewater reuse in agricultural systems. Recent studies highlight the pivotal role of soil microbial communities in mediating these processes [11], as their diversity, composition, and functional traits [12] are influenced by wastewater and microalgae irrigation. For instance, certain bacteria including *Enterobacter*, *Flavisolibacter*, *Adhaeribacter*, and *Bacillus* have been known to enhance plant growth [13,14].

The diversity and composition of rhizosphere soil bacterial community are strongly affected by the soil environment. Elevated soil carbon content can alleviate carbon deficiency stress on microbial growth, consequently promoting the proliferation and development of bacterial community [10]. However, the impacts of nitrogen and salinity on bacterial communities remain unclear [15,16]. Compared to nitrogen, phosphorus enrichment exhibits a relatively minor impact on soil microbial community composition [17]. Elevated nitrogen concentrations may enhance carbon and phosphorus availability [18] and improve soil salt metabolism capacity [19]. The combined application of nitrogen and phosphorus can change the soil pH, thereby indirectly altering bacterial community [20]. Notably, soil pH has been shown to exhibit a highly significant correlation with bacterial community composition [21].

Correspondingly, rhizosphere bacteria can influence plant growth and soil environment [22] through multiple biochemical pathways, including mediating soil nitrogen transformation, synthesizing exopolysaccharides, and releasing volatile organic compounds [14]. For example, Siddikee et al. [23] found that *Phomopsis liquidambari* could promote rice growth by enhancing the functional diversity of microbial communities. Liu et al. [18] discovered functional genes in *S. salsa* rhizosphere bacteria associated with salt stress adaptation, nutrient cycling regulation, and plant growth promotion. Furthermore, Li et al. [24] revealed nitrogen metabolic pathways in soil microorganisms and the relationship between the associated functional genes and environmental changes within the soil.

Interactions among plants, soil, and bacteria are crucial for nutrient cycling [20]. However, the effects of wastewater and microalgae irrigation on plant growth, soil nutrients, and bacterial community structure–function relationships in saline–alkali ecosystems remain insufficiently characterized. Therefore, we selected *S. salsa* and its rhizosphere soil as the research subjects to systematically evaluate plant–soil systems irrigated under different irrigation treatments. This research aimed to (1) examine the growth of *S. salsa* and changes in rhizosphere soil physicochemical properties, (2) analyze the composition of rhizosphere bacterial communities and the relationship between them and soil environmental factors, and (3) reveal the ecological function of bacterial communities in the rhizosphere soil of *S. salsa*.

## 2. Materials and Methods

### 2.1. Soil Sample and Irrigation Water Preparation

Saline soil was collected from Huyanghe City (44°20′–47°04′ N, 83°51′–85°51′ E) in Xinjiang Uygur Autonomous Region, China. The saline wastewater used for irrigation was obtained from the effluent of a municipal wastewater treatment plant in Huyanghe City, and it contained 1.18 ± 0.02 mg/L ${\mathrm{NH}}_{4}^{+}$-N, 0.26 ± 0.02 mg/L ${\mathrm{NO}}_{3}^{-}$-N, 0.02 ± 0.00 mg/L ${\mathrm{PO}}_{4}^{3-}$-P, 0.03 ± 0.00 mg/L total phosphorus (TP), and 70.56 ± 3.46 mg/L chemical oxygen demand (COD), with an electrical conductivity (EC) of 5.76 ± 0.20 mS/cm. The microalgae *Tetradesmus obliquus* ZYY1 was cultured in BG11 medium (the specific composition is described in Text S1), stirred at a speed of 200 rpm at 30 °C, and provided continuous illumination of 2000 lux, all under injection of 2.5% CO_2_. During the logarithmic growth stage, the microalgae were harvested and centrifuged at 5000 rpm (TGL-18000CR, Anting Scientific Instrument Factory, Shanghai, China). Subsequently, the resulting biomass was washed three times with distilled water. Finally, the microalgal biomass was added to the saline wastewater to achieve a final microalgal concentration of 0.05 g/L.

### 2.2. Experimental Design

A 100-day pot experiment was conducted in August 2023 in Shihezi City (44°15′–44°19′ N, 85°59′–86°08′ E), Xinjiang Uygur Autonomous Region, China. The outdoor temperature and humidity ranged from 10 °C to 35 °C and 30% to 45%, respectively, with a 24 h photoperiod and light intensity of 2000 lux. Each pot (with a bottom diameter of 24 cm, a top diameter of 28 cm, and a height of 30 cm) was filled with 20 kg of soil. Six seeds were sown into each pot and irrigated every five days; the amount of irrigation was adjusted according to changes in evaporation. Three kinds of irrigation water were set up for the experiment: fresh water (FW), saline wastewater (SW), and saline wastewater with microalgae (SWM). There were three replicates for each treatment. At the end of the experiment, soil clinging to the roots was gently removed with a soft sterile brush to obtain rhizosphere soil. Plant growth parameters (height of the plant, diameter of the roots, and total dry weight) were measured. The collected soil samples were sieved to pass through a 2 mm mesh to remove impurities to analyze rhizosphere soil properties, physicochemical characteristics, and bacterial community composition.

### 2.3. Measurement of the Biomass of S. salsa and Rhizosphere Soil Properties

The plant height and root diameter of the *S. salsa* were measured with digital calipers (±0.01 mm precision), and the roots were then rinsed with distilled water and dried at 70 °C for 48 h. Subsequently, the plants were weighed to obtain the total dry weight. The soil pH and electrical conductivity (EC) were measured in 1 (soil): 5 (water) (*w*/*v*) suspension using a pH and conductivity meter. Salt content was determined by the gravimetric method, total nitrogen (TN) was determined via the Kjeldahl method, available nitrogen (AN) was assessed using the alkali-hydrolyzed diffusing method, and available phosphorus (AP) was measured by the sodium hydrogen carbonate solution-Mo-Sb anti spectrophotometric method. The flame photometry method was used to analyze available potassium (AK), and soil organic carbon (SOC) was determined through potassium dichromate oxidation titration [1].

### 2.4. Analysis of Rhizosphere Soil Bacterial Communities

Bacterial DNA was extracted from soil samples using the E.Z.N.A.^®^ Soil DNA Kit (Omega Bio-Tek, Norcross, GA, USA). The V3–V4 region of the bacterial 16S rRNA gene was amplified using primers 341F and 806R, then, the amplicon library was paired-end sequenced on an Illumina MiSeqPE250 platform (Illumina, San Diego, CA, USA) by Shanghai BIOZERON Co., Ltd. (Shanghai, China). The raw reads were deposited into the NCBI Sequence Read Archive database (Accession Number: PRJNA1199149). The original DNA data were processed using Qiime2 (for quality control, filtering, and removal of chimeras), and amplicon sequence variants (ASVs) were determined using the DADA2 plugin in Qiime2. Alpha-diversity, beta-diversity, community composition, biomarker discovery, environmental factor correlations, and 16S rRNA gene function were analyzed.

### 2.5. Data Processing and Statistical Analysis

The statistical analyses of experimental data were performed using Microsoft Excel 2016 (Microsoft Corp., Redmond, WA, USA) and SPSS ver. 20.0 (IBM Corp., Armonk, NY, USA). The least significant difference (LSD) test (*p* < 0.05) was performed to control for multiple comparisons of the samples, and data are represented as mean ± standard deviation (SD) values herein.

## 3. Results

### 3.1. Suaeda Salsa Growth

**Figure 1:**
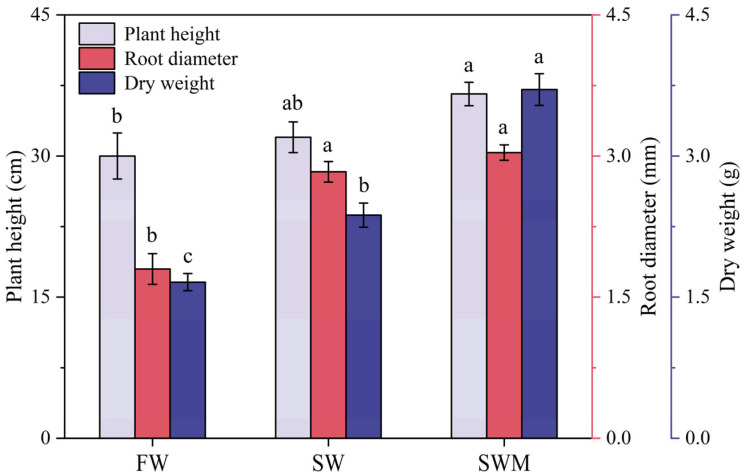
Mean (±SD) growth of *Suaeda salsa* under the different irrigation water treatments. The different lowercase letters indicate significant differences between treatments according to the least significant difference (LSD) test (*p* < 0.05).

Plant height, root diameter, and total dry weight under the SW and SWM irrigation treatments were significantly increased compared with those under the FW irrigation treatment (*p* < 0.05) (Figure 1), demonstrating that *S. salsa* exhibits salt tolerance and can physiologically adapt to moderate saline environments. After the addition of microalgae, the SWM irrigation treatment showed a significantly higher plant height (14.6%), root diameter (7.2%), and dry weight (56.4%) compared to the SW irrigation treatment (*p* < 0.05), reaching 36.67 ± 1.67 cm, 3.03 ± 0.30 mm, and 3.71 ± 0.17 g, respectively (Figure 1).

### 3.2. Soil Physicochemical Properties

ANOVA results indicated that irrigation water significantly influenced all soil physicochemical properties (Table 1). Compared with the group without *S. salsa* (original soil, OS), the cultivation of *S. salsa* significantly reduced soil pH value by 0.9–3.8% and increased the contents of TN, AK, and SOC by 37.04–48.15%, 52.23–58.69%, and 95.04–142.98%, respectively. Under saline water irrigation, soil pH value, and contents of TN, AN, AP, and SOC in the soil were 1.87–3.00%, 5.41–8.11%, 32.82–39.26%, 13.81–39.20%, and 0.42–24.58% higher than those under FW irrigation, respectively. Each of these parameters showed a significant increase following microalgae application. The EC and salt content of rhizosphere soil under FW and SW irrigation were significantly higher compared with those in the OS group. The addition of microalgae significantly decreased the salt content (7.74 ± 0.08 g/kg) and EC value (2.03 ± 0.03 mS/cm) of soil (*p* < 0.05), which decreased by 6.63–13.52% and 5.58–21.62%, respectively (Table 1).

### 3.3. Soil Bacterial Community Diversity

In this study, we obtained 34,671, 39,938, 34,600, and 34,587 valid sequences from soil samples of the OS, FW, SW, and SWM groups, respectively (Table 2). Among these sequences, 99.08% were 401–500 bp, with average lengths of 417.91, 415.35, 415.51, and 416.82 bp for the OS, FW, SW, and SWM groups, respectively (Figure S1a). Both rarefaction curves and Shannon–Wiener indices plateaued with increasing sequencing depth (Figure S1b,c), indicating sufficient sampling coverage to characterize bacterial diversity in each sample. Richness index (observed species, Chao1, ACE) and diversity index (Shannon, Simpson, Pielou_J and Pd_faith) were used to assess the alpha diversity of soil bacteria (Table 2). The FW irrigation samples exhibited the highest observed species, Chao1, ACE, Shannon, and Pielou_J, index values, of 1244.00, 1244.08, 1244.88, 6.50, and 0.9115, respectively, which indicates that the FW irrigation treatment had the highest microbial richness, diversity, and evenness. In contrast, the observed species (955.00), Chao1 (955.00), ACE (955.19), Shannon (6.12), Pielou_J (0.8918), and Pd_faith (59.83) index values were the lowest under SW irrigation, with decreases of 23.23%, 23.24%, 23.27%, 5.85%, 2.16%, and 14.88%, respectively, compared with the FW irrigation treatment, indicating that saline water irrigation significantly reduced the diversity and evenness of soil microbial communities. Compared to the SW irrigation treatment, the SWM treatment with the addition of microalgae had values of the observed species, Chao1, ACE, Shannon, and Pd_faith index values of 1160.00, 1160.00, 1160.00, 6.29, and 74.93, respectively, increases of 21.47%, 21.47%, 21.44%, 2.78%, 0.01%, and 20.15%, respectively. Notably, the SWM treatment exhibited the highest Pd_faith index value, indicating that microalgae application enhanced phylogenetic diversity.

Beta diversity was evaluated using Venn diagrams and principal coordinates analysis (PCoA) (Figure S2), revealing the connections, commonalities, and distinctions among the studied soil sample treatments (OS, FW, SW, and SWM). The Venn diagrams represent the number of common and unique ASVs across multiple samples, revealing a total of 2995 ASVs shared among all groups. The OS, FW, SW, and SWM groups contained 978, 1266, 962, and 1182 ASVs, respectively, with 161 ASVs shared across all groups, accounting for 16.5% (OS), 12.7% (FW), 16.7% (SW), and 13.6% (SWM) of ASVs in each group, respectively. The OS, FW, SW, and SWM groups had 438, 654, 394, and 677 unique ASVs, respectively, underscoring the distinct structure of bacterial communities under different treatments. The PCoA results indicated that the first principal component accounted for 87% of the total variance, and the contribution rates of the first and second axes were 61% and 26%, respectively. All four sample treatments clustered separately, accordingly, the planting of *S. salsa*, saline water irrigation, and microalgae addition had significant effects on the bacterial community structure of rhizosphere soil.

### 3.4. Soil Bacterial Community Composition

**Figure 2:**
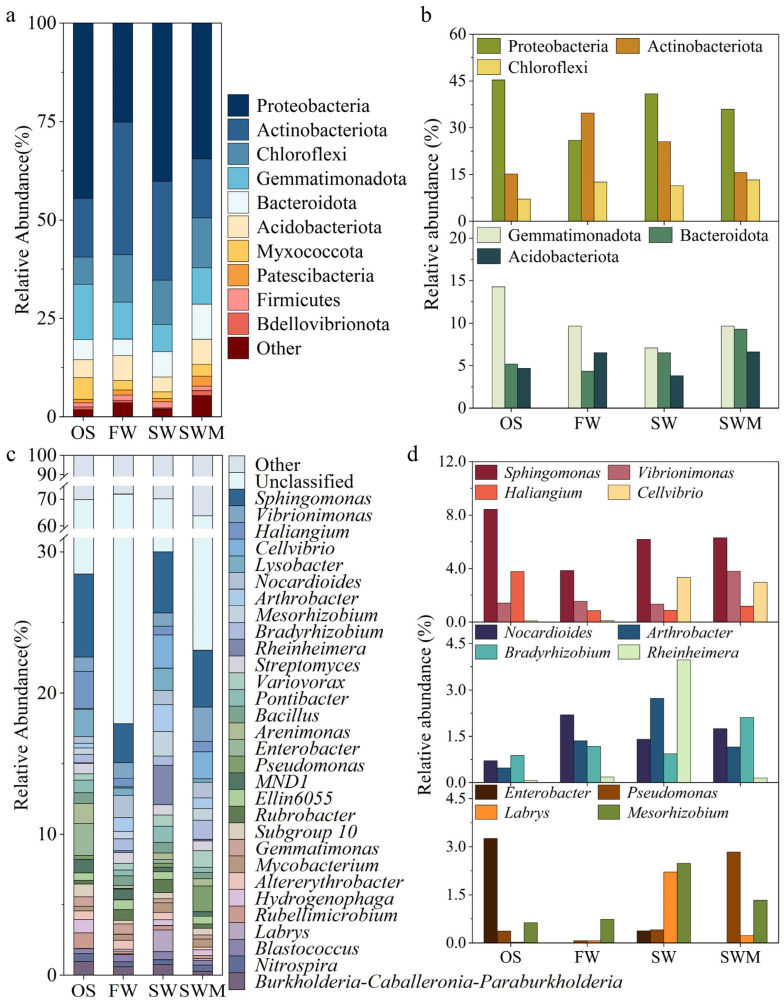
The relative abundances of (**a**) the top 10 most common bacterial phyla and (**b**) bacterial phyla with significant differences among treatments; (**c**) the relative abundances of the top 30 most common bacterial genera and (**d**) bacterial genera with significant differences among treatments.

A total of 33 phyla were identified in the samples, including some unclassified bacteria (Figure 2a). Among these, the 10 phyla with the highest relative abundances were considered the dominant phyla, and their relative abundances varied among samples. The bacterial communities across all treatments were predominantly composed of Proteobacteria (25.14–44.51%), Actinobacteriota (14.90–33.61%), Chloroflexi (6.91–12.69%), Gemmatimonadota (6.95–14.02%), Bacteroidota (4.20–8.91%), and Acidobacteriota (3.75–6.34%). These core phyla collectively accounted for over 85% of the total bacterial sequences in all treatments. Figure 2b further illustrates the responses of soil bacterial communities to different irrigation treatments. Compared to the OS group, rhizosphere soils of *S. salsa* exhibited higher relative abundance of Chloroflexi. The SW irrigation increased the relative abundance of Proteobacteria and Bacteroidota, while decreasing the relative abundance of Actinobacteriota. In contrast, the SWM treatment increased the relative abundance of Chloroflexi (12.69%), Gemmatimonadota (9.25%), Bacteroidetes (8.91%), and Acidobacteriota (6.34%) phyla, while reducing the abundance of Proteobacteria and Actinobacteriota phyla. Notably, the SWM treatment showed the highest relative abundances of both Chloroflexi and Bacteroidota among all four treatments.

Bacterial genera with a relative abundance in the top 30 are shown in Figure 2c. Among these, *Sphingomonas* (2.78–5.89%) was the most prevalent genus across all soil samples. Compared to the other treatments, *Sphingomonas* (5.89%), *Haliangium* (2.64%), and *Enterobacter* (2.28%) were the dominant groups in the OS group (Figure 2c,d). The cultivation of *S. salsa* (FW, SW, and SWM treatments) significantly increased the relative abundances of the bacterial genera *Cellvibrio* (0.07–2.35%), *Nocardioides* (0.99–1.58%), *Arthrobacter* (0.74–1.92%), *Bradyrhizobium* (0.66–1.36%), *Rheinheimera* (0.09–0.13%), and *Mesorhizobium* (0.53–1.74%). Compared to FW irrigation, SW and SWM irrigation treatments significantly increased the relative abundances of *Sphingomonas*, *Cellvibrio*, *Labrys*, *Enterobacter*, *Pseudomonas*, and *Mesorhizobium*. Notably, the addition of microalgae (SWM irrigation) promoted higher abundances of *Sphingomonas*, *Vibrionimonas*, *Haliangium, Nocardioides, Bradyrhizobium*, and *Pseudomonas* compared to SW irrigation.

**Figure 3:**
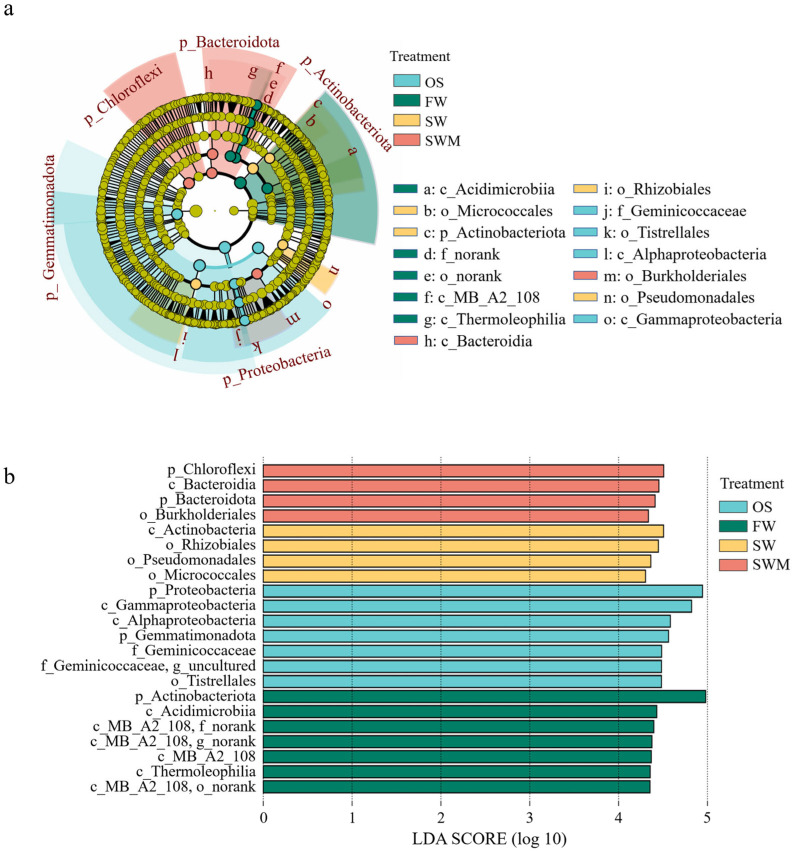
(**a**) The identified hierarchical taxonomic structure and (**b**) linear discriminant analysis (LDA) of the bacterial composition in rhizosphere soil based on linear discriminant effect size (LEfSe) analysis (LDA > 4, *p* < 0.05).

To further analyze and identify the varying characteristics of bacterial communities among treatments, the species with significant differences among different groups (i.e., biomarkers) were analyzed by linear discriminant effect size (LEfSe) analysis (Figure 3). There were 15 distinct bacteria with varied abundances between the OS samples and the rhizosphere soil of *S. salsa* samples under the three irrigation treatments (LDA > 4, *p* < 0.05). The biomarkers in the OS samples included f_Geminicoccaceae (o_Tistrellales, c_Alphaproteobacteria, p_Proteobacteria), c_Gammaproteobacteria (p_Proteobacteria), and p_Gemmatimonadota. The biomarkers of FW irrigation consisted of c_MB-A2-108 (p_Actinobacteriota), c_Acidimicrobiia (p_Actinobacteriota), and c_Thermoleophilia (p_Actinobacteriota). Under the SW irrigation treatment, o_Rhizobiales (c_Alphaproteobacteria, p_Proteobacteria), o_Micrococcales (c_Actinobacteria, p_Actinobacteriota), and o_Pseudomonadales (c_Gammaproteobacteria, p_Proteobacteria) were more abundant. Under the SWM irrigation treatment, o_Burkholderiales (c_Gammaproteobacteria, p_Proteobacteria), c_Bacteroidia (p_Bacteroidota), and p_Chloroflexi were more abundant. Thus, there were notable differences in bacterial community composition associated with soil, *S. salsa*, and irrigation water.

### 3.5. The Correlation Between Soil Bacterial Communities and Soil Physicochemical Properties

**Figure 4:**
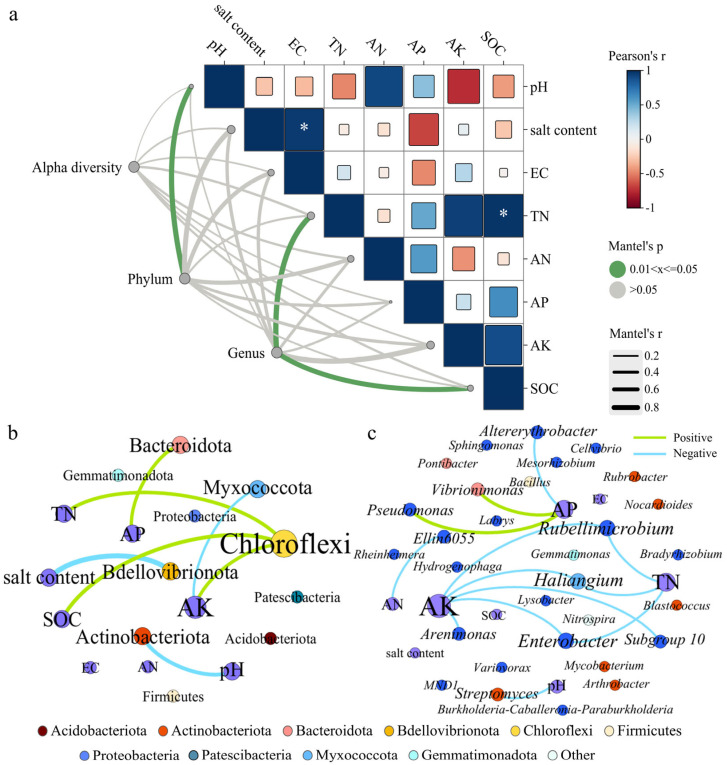
(**a**) The Mantel test between bacterial community and environmental factors. Note: Alpha diversity is represented by the number of sequences, amplicon sequence variants (ASVs), and Observed_species, Chao1, ACE, Shannon, Simpson, Pielou_J and Pd_faith index values of the bacterial community. * Indicates that the correlation of the environmental factor is significant at *p* < 0.05. The co-occurrence networks show the interaction between environmental factors and (**b**) phyla (**c**) and genera.

In this study, Mantel tests were employed to evaluate associations between bacterial community structure (including α diversity, dominant phyla, and genera) and environmental factors across the four treatment groups (Figure 4a). SOC was positively correlated with TN (Table S1), and both exhibited their highest contents under the SWM irrigation treatment. Although soil environmental factors exerted no significant influence on the α diversity of bacteria (Table S2), and pH significantly influenced phyla composition (*p* < 0.05), while TN and SOC were determinants of genus community structure.

Saline water irrigation and microalgae addition significantly altered the relationships between rhizosphere soil microbial communities and soil physicochemical properties. To further explore these interactions, the relationships were evaluated as shown in Figure 4b,c. In Figure 4, node size corresponds to connection degree; green/blue edges represent positive/negative correlations, respectively. Figure 4b,c display 10 phyla and 30 genera, revealing a total of 20 significant correlations; the specific values are shown in Tables S3 and S4. Actinobacteriota abundance was negatively correlated with pH, while Bacteroidota correlated positively with AP. Chloroflexi showed highest connectivity in Figure 4b, and its abundance was positively correlated with the contents of TN, AK, and SOC.

In Figure 4c, AK exhibits the most connections, followed by AP and TN. AK and TN were negatively correlated with abundances of *Haliangium*, *Enterobacter*, and *Rubellimicrobium*. Additionally, AK and AN were negatively correlated with abundances of *Arenimonas* and *Ellin*6055. AP showed positive correlations with *Vibrionimonas* and *Pseudomonas* but negative correlation with *Altererythrobacter*.

### 3.6. Soil Bacterial Community Function

#### 3.6.1. Metabolic Pathways Based on PICRUSt2

**Figure 5:**
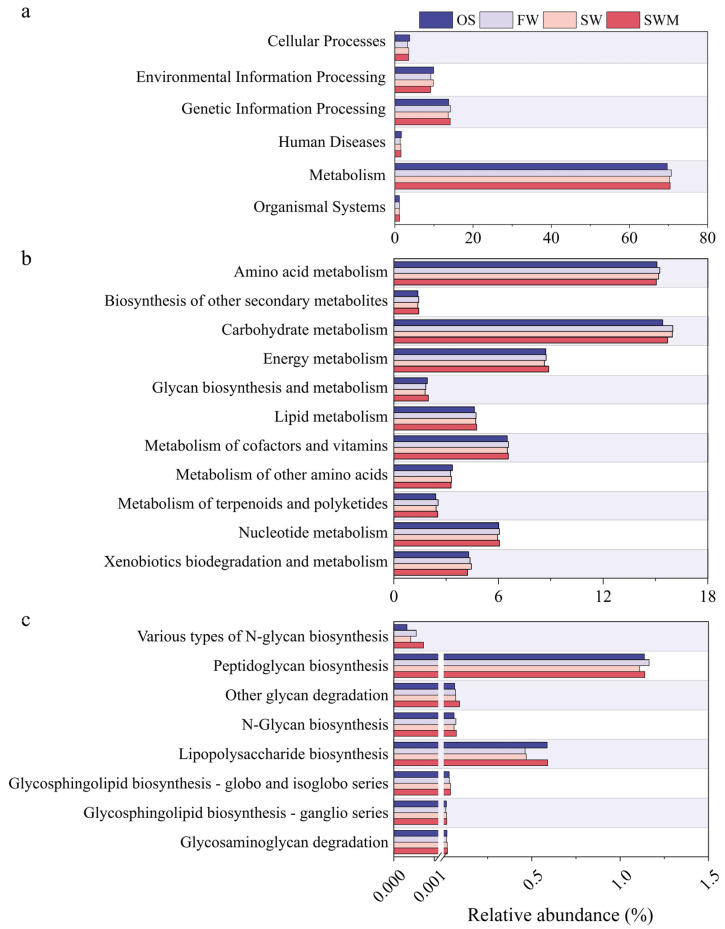
Function profiles of bacteria at (**a**) level 1, (**b**) level 2 (metabolism), and (**c**) level 3 (glycan biosynthesis and metabolism) based on PICRUSt.

Figure 5 shows analysis of biological metabolic pathways based on PICRUSt2, which can help reveal the potential benefits of the bacterial community. Metabolic pathways are presented at three levels. At level 1 (Figure 5a), metabolism (69.69–70.75%) was the main functional classification, followed by genetic information processing (13.64–14.25%), environmental information processing (9.13–9.92%), cellular processes (3.28–3.80%), human diseases (1.35–1.67%), and organismal systems (1.13–1.19%). Since most of these genes were determined to be involved in metabolic processes, those specifically connected to metabolism were further analyzed at level 2 (Figure 5b). The relative abundance of carbohydrate metabolism (15.70–15.99%) and metabolism of cofactors and vitamins (6.51–6.58%) in the rhizosphere soil were higher compared to the OS treatment. In the soil under SWM irrigation, the bacterial communities involved in energy metabolism (8.88%) and metabolism of cofactors and vitamins (6.56%) showed higher abundances compared to SW irrigation. Sugar synthesis represents a critical functional trait that enhances plant stress tolerance. Therefore, we compared the relative abundances of related genes (Figure 5c). Distinct from OS soil, *S. salsa* rhizosphere soils exhibited enriched genes associated with glycosphingolipid biosynthesis-globo and isoglobo series, N-glycan biosynthesis, other glycan degradation, peptidoglycan biosynthesis, and various types of N-glycan biosynthesis. Nearly all genes related to glycan synthesis were more abundant in SW and SWM irrigation treatments than FW treatment (Figure 5c). However, genes involved in peptidoglycan biosynthesis were slightly less abundant under SW and SWM irrigation treatments than under FW irrigation. Notably, all genes involved in sugar synthesis under SWM treatment showed higher abundance than those under SW treatment. Additionally, the enrichment of two-component systems was found in OS and SW irrigation samples (Table S5). Nitrogen metabolism is related to plant growth, with the highest abundance under SWM irrigation, followed by SW irrigation, FW irrigation, and OS treatment, indicating that the metabolic processes of bacterial communities are enhanced by *S. salsa* cultivation, saline water (SW and SWM) irrigation, and microalgae addition. These functional analyses provide key insights into how soil bacteria support *S. salsa* health and soil ecosystem resilience.

#### 3.6.2. Bacterial Community Functional Predictive Based on FAPROTAX

**Figure 6:**
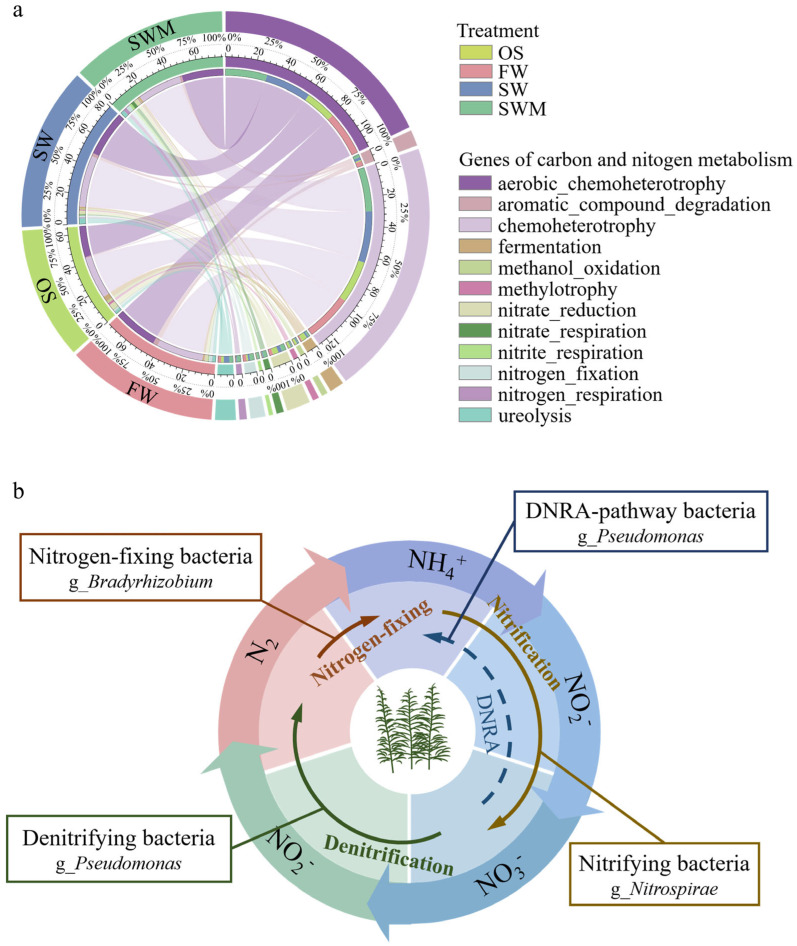
(**a**) Circos plot showing the differences in carbon and nitrogen metabolism-related functions among the four treatments and (**b**) the dominant bacterial community in the rhizosphere soil were predicted to participate in the nitrogen cycle under SWM irrigation based on FAPROTAX analysis.

Functional predictions of bacterial communities from the four treatment groups based on the FARPROTAX database (top 30) are shown in Table S6. Chemoheterotrophy (28.82–29.89%), aerobic_chemoheterotrophy (21.04–31.30%), fermentation, and processes related to nitrogen metabolism, including nitrate_reduction, ureolysis, nitrogen_fixation, nitrate_respiration, and nitrogen_respiration, were dominant among the four groups of samples, each having a relative abundance exceeding 1%. Figure 6a shows the differences in carbon and nitrogen metabolism-related functions among the four treatments. Compared with OS samples, the functional abundance of genes related to chemoheterotrophy, aerobic_chemoheterotrophy, nitrogen_fixation, and aromatic_compound_degradation within the rhizosphere bacterial community of the *S. salsa* rhizosphere was increased by more than 1%. Furthermore, compared with FW, SW and SWM irrigation treatments enhanced the functional abundance of genes related to nitrogen metabolism (nitrate_reduction, ureolysis, nitrate_respiration, nitrogen_respiration and nitrite_respiration), fermentation, methylotrophy-related (methylotrophy, methanol_oxidation), and phototrophy. In addition, compared with SW irrigation, the relative abundance of nitrogen metabolism related genes (i.e., nitrate_reduction, nitrogen_fixation, nitrate_respiration, nitrogen_respiration, and nitrite_respiration) increased under SWM irrigation. These results indicated that planting *S. salsa* and saline water (SW and SWM) irrigation differentially enhances microbial functions associated with carbon cycling and nitrogen metabolism, with microalgae addition further accelerating soil nitrogen cycling.

## 4. Discussion

### 4.1. Physicochemical Properties of the Rhizosphere Soil

The improvement of soil quality is typically reflected in the enhancement of soil biochemical properties. Our findings demonstrate that irrigation water significantly affects soil physicochemical characteristics (Table 1). Among the four samples, significant variations in pH values were observed. Specifically, the cultivation of *S. salsa* (FW) led to a decrease in soil pH. In contrast, saline water irrigation resulted in significantly higher pH values compared to the FW treatment, and there was a further increase following microalgae application. Previous research has demonstrated the significant capacity of *S. salsa* for reducing soil alkalinity [20]. The pH elevation observed under SWM irrigation could potentially be attributed to the presence of a large amount of organic matter in microalgae, which is decomposed by microorganisms and released as organic anions [25].

In terms of soil salinity regulation, the halophyte *S. salsa* exhibits considerable capacity. However, our results showed elevated EC and salt content in rhizosphere soil under FW irrigation compared to the OS group. This observation may be attributed to the salt accumulation properties of *S. salsa*, consistent with the findings of Zhang et al. [20]. Notably, microalgae application significantly decreased soil salinity parameters, likely through the chelation of salt ions by extracellular polymeric substances produced by microalgae [26].

Regarding soil nutrient cycling, our study demonstrated that the cultivation of *S. salsa* alone significantly elevated concentrations of TN, AK, and SOC. These enhancements likely stem from root exudates and plant litter, which enhance carbon and TN accumulation in the rhizosphere soil [20]. Further investigation demonstrated that saline water irrigation exhibited significantly higher levels of TN, AN, AP and SOC compared to the FW treatment. Furthermore, these parameters showed additional increases following microalgae application. These findings suggest that wastewater possesses substantial potential as a nutrient source. Consistent with our observations, Dineshkumar et al. [27] found significant increases in soil AN, AP, and AK soils amended with microalgae. As an organic fertilizer, microalgae mitigate nutrient loss by gradually releasing nitrogen, phosphorus, and potassium into the soil. Previous research has also established the influence of fertilizer application on soil organic carbon accumulation [22]. In conclusion, these results demonstrate that *S. salsa* grown in saline environments and microalgae addition contribute to soil desalination while increasing soil nitrogen, phosphorus, and carbon content, resulting in marked improvements in overall soil fertility.

### 4.2. Diversity and Composition of Bacterial Community

Soil microbial diversity serves as a critical bioindicator of soil quality and reflects the overall dynamics of microbial communities [28]. In this study, FW irrigation maintained significantly higher bacterial community diversity and richness than other samples. As a halophytic species, *S. salsa* has been demonstrated to enhance microbial activity in saline soils and promote the healthy development of saline–alkali land ecosystems [7]. However, saline water irrigation significantly decreased both α diversity indices of soil microbial communities. The impact of salinity on soil microbial diversity remains inconclusive in the literature [29], and the study results indicate that saline irrigation may selectively inhibit the growth of certain bacterial taxa.

PCoA revealed that *S. salsa* cultivation, saline water irrigation, and microalgae application significantly influenced the rhizosphere bacterial community structure. These structural changes were likely owing to the fact that plant roots can secrete hormones that affect the rhizosphere soil bacterial community [20]. In addition, the bacterial community under the addition of microalgae showed the greatest difference compared to other treatment groups, indicating its significant role in ecosystem functioning [30]. Therefore, it can be reasonably concluded that microalgae can alter the rhizosphere soil bacterial community structure.

At the phylum level, Proteobacteria and Actinobacteriota were the dominant bacterial phyla in the rhizosphere soil, and these phyla are common in other saline soil environments and the rhizosphere of halophytes [22]. It has been shown that the abundance of Actinobacteriota is significantly reduced in alkaline soils compared to neutral pH soils [31]. Furthermore, both Actinobacteriota and Proteobacteria demonstrate ecological adaptations to low-carbon environments [10,32]. This may explain the observed increase in Proteobacteria relative abundance and decrease in Actinobacteriota relative abundance under saline water irrigation, as well as the further decline in both phyla following microalgae application. In the rhizosphere soil of *S. salsa* (FW, SW, and SWM treatments), the relative abundance of Chloroflexi was significantly elevated relative to the OS group, and this finding aligns with previous studies showing that Chloroflexi abundance is positively correlated with soil organic matter content [33]. Saline water irrigation significantly increased the relative abundance of Bacteroidota, consistent with its positive correlation with nutrient availability [10]. SWM irrigation elevated Gemmatimonadota abundance, and enriched both the abundance of Chloroflexi and Bacteroidetes, suggesting that microalgae addition enhanced the availability of carbon and nitrogen sources in the rhizosphere soil, consequently promoting the proliferation of these three bacterial phyla [34].

At the genus level, the OS group demonstrated significant enrichment of halotolerant genera, including *Sphingomonas*, *Haliangium*, and *Enterobacter* [14,18]. Following *S. salsa* cultivation, the abundance of aerobic or strictly aerobic bacteria significantly increased, including *Cellvibrio*, *Nocardioides*, *Arthrobacter*, *Bradyrhizobium*, *Rheinheimera*, and *Mesorhizobium*. This condition can be attributed to the fact that the majority of these bacteria are aerobic or strictly require O_2_, and soil moisture impacts the amount of O_2_ available, creating distinct microenvironments that select for specific aerobic bacterial communities [18]. The abundance of *Pseudomonas*, a genus with denitrification potential, significantly increased under saline water irrigation and reached its peak level following microalgae application [33]. The addition of nitrogen also led to an increase in the relative abundance of denitrifying bacteria in *S. salsa* rhizosphere soil [35]. In addition to *Pseudomonas*, the SWM treatment was enriched with multiple bacterial genera, including *Sphingomonas*, *Vibrionimonas*, *Nocardioides*, and *Bradyrhizobium*. Studies have shown that variations in soil nitrogen and phosphorus contents can alter root exudates, consequently supporting the growth of different bacterial species [18]. Furthermore, biochar supplementation has been shown to enhance the proliferation of phosphate-solubilizing bacteria through improved microhabitat conditions, such as *Pseudomonas* [36].

### 4.3. Influence of Soil Physicochemical Properties on Bacterial Community Composition

Our study revealed that rhizosphere soil bacterial communities constitute an intricate interaction network. This network is influenced by multiple factors, especially interactions with soil physicochemical properties. Under SWM irrigation, both SOC and TN reached their highest levels and exhibited a significant positive correlation. Previous studies have shown that soil with high TOC content indicated ecosystem health [37]. Additionally, the impact of soil environmental factors on α-diversity indices was not significant, suggesting that soil bacterial communities maintain high stability under environmental changes [10]. Rhizosphere microbial composition exhibited significant associations with three key soil parameters: pH, TN, and SOC. These findings are strongly supported by the study of [38], who similarly identified pH, TN, and SOC as dominant drivers of bacterial community structure.

In vegetated saline–alkaline ecosystems, Actinobacteriota serve vital ecological functions. Our results revealed a significant negative correlation between Actinobacteriota abundance and soil pH, consistent with prior observations of their reduced abundance in alkaline versus neutral soils [31]. Furthermore, Bacteroidetes abundance was positively correlated with AP, while Chloroflexi showed positive correlations with TN, AK, and SOC, aligning with the results of Hao et al. and Wang et al. [38,39].

In this study, nitrogen exhibited strong correlations with specific bacterial genera. *Haliangium*, *Enterobacter*, and *Rubellimicrobium* were negatively correlated with TN, while *Ellin6055* showed a negative correlation with AN. *Haliangium* is a bacterial genus with denitrifying functions [40]. Both *Enterobacter* and *Rubellimicrobium* belong to Proteobacteria, which exhibit a significant negative correlation with soil nitrogen content [16]. *Ellin*6055 is associated with nitrite oxidation [40]. An increase in the abundances of *Haliangium* and *Ellin6055* may promote the conversion of ${\mathrm{NO}}_{3}^{-}$ and ${\mathrm{NO}}_{2}^{-}$ in the soil into N_2_, potentially contributing to nitrogen loss from the system. Additionally, the relative abundance of *Vibrionimonas* and *Pseudomonas* correlated positively with AP content, while *Altererythrobacter* showed a negative correlation with AP. Both *Vibrionimonas* and *Altererythrobacter* participate in phosphorus cycling [28,41]. *Pseudomonas* is a common phosphate-solubilizing microorganism, which can increase the soil AP content [42]. *Streptomyces* abundance was negatively correlated with pH in this study, and this genus has the ability to change environmental pH [43].

### 4.4. Functional Prediction of Rhizosphere Soil Bacteria

Functional prediction of bacterial communities was performed using PICRUSt2 based on 16S rRNA gene sequencing data. The predicted results indicated that carbohydrate metabolism was the predominant metabolic pathway across all samples. The *S. salsa* rhizosphere soil exhibited significantly higher carbohydrate metabolism gene abundance compared to the OS group, and carbohydrate metabolism generally enhances plant stress resistance [6]. The SWM irrigation treatment significantly enriched bacterial communities related to energy metabolism, metabolism of cofactors and vitamins, suggesting that more ester compounds, soluble sugar, and other osmotic substances occurred in soil with added microalgae, thereby increasing their energy metabolism [44]. Additionally, soluble sugar accumulation may play a critical role in plant salt tolerance [45]. Notably, the higher abundance of two-component systems in OS and SW irrigation samples may help bacterial communities adapt to stressful environmental conditions [46].

The nitrogen cycle is a vital component of the soil nutrient cycle. PICRUSt2 and FAPROTAX analyses showed significant enrichment of nitrogen metabolism-related genes in SWM samples. The application of SWM irrigation led to the notable enrichment of functional bacteria involved in nitrogen transformation processes (Figure 2c). Specifically, *Bradyrhizobium*, which is associated with nitrogen fixation [47], and *Pseudomonas*, which is capable of performing denitrification as well as converting nitrate to ammonium through dissimilatory reduction [48]. The nitrifying bacteria *Nitrospira* mediates aerobic nitrite oxidation, consistent with its lower abundance relative to OS conditions under SWM irrigation, but it still contributed to soil nitrogen accumulation and benefited *S. salsa* growth. Based on the analysis of the nitrogen transformation process of the bacterial community participating under SWM irrigation mentioned above, a method of remediation is suggested (Figure 6b). The elevated nitrogen content in the rhizosphere soil following microalgae addition indicates a close relationship between microbial community composition and enhanced nitrogen cycling activity (Table 1). This enrichment plays a crucial role in the foundation for nitrogen accumulation in the soil under SWM irrigation, providing a theoretical foundation for saline–alkali soil remediation and ecosystem restoration.

## 5. Conclusions

The addition of microalgae reduced the salinity and improved the nutrient content in the soil, and altered the diversity and composition of the rhizosphere soil bacterial community. The soil rhizosphere bacteria exhibited a greater abundance of salt stress defense and nitrogen metabolism-related genes, especially microalgae, enriched the nitrogen metabolism of bacteria, and subsequently promoted the growth of *S. salsa*. This study highlights the community formation and functional differences in rhizosphere bacterial communities of *S. salsa* under different irrigation treatments and lays a foundation for the possible utilization of microalgae to promote plant growth and improve saline alkali land.

## Figures and Tables

**Table 1:** Soil physicochemical properties.

Variable	Treatment			
	OS	FW	SW	SWM
pH	7.63 ± 0.01 ^a^	7.34 ± 0.03 ^d^	7.48 ± 0.02 ^c^	7.56 ± 0.02 ^b^
salt (g/kg)	8.29 ± 0.07 ^b^	8.32 ± 0.03 ^b^	8.95 ± 0.24 ^a^	7.74 ± 0.08 ^c^
EC (mS/cm)	2.15 ± 0.04 ^b^	2.22 ± 0.03 ^b^	2.59 ± 0.06 ^a^	2.03 ± 0.03 ^c^
TN (g/kg)	0.27 ± 0.00 ^d^	0.37 ± 0.00 ^b^	0.39 ± 0.00 ^c^	0.40 ± 0.00 ^a^
AN (mg/kg)	11.53 ± 0.30 ^a^	8.38 ± 0.13 ^c^	11.13 ±0.29 ^b^	11.67 ± 0.17 ^ab^
AP (mg/kg)	10.69 ± 0.20 ^bc^	9.85 ± 0.10 ^c^	11.21 ± 0.26 ^b^	16.20 ± 0.54 ^a^
AK (mg/kg)	122.88 ±1.15 ^c^	195.00 ± 2.84 ^a^	190.09 ± 2.83 ^ab^	187.06 ± 0.57 ^b^
SOC (g/kg)	1.21 ± 0.00 ^d^	2.36 ± 0.02 ^c^	2.37 ±0.03 ^b^	2.94 ± 0.01 ^a^

Data are presented as mean ± standard deviation values (n = 3). The different lowercase letters indicate significant differences between treatments (least significant difference, *p* < 0.05).

**Table 2:** Alpha diversity indices of soil bacterial community.

Alpha Diversity	Treatment			
	OS	FW	SW	SWM
No. of sequences	34,671	39,938	34,600	34,587
ASVs	978	1266	862	118
Observed_species	976.00	1244.00	955.00	1160.00
Chao1	976.00	1244.08	955.00	1160.00
ACE	976.00	1244.08	955.19	1160.00
Shannon	6.16	6.50	6.12	6.29
Simpson	0.9960	0.9969	0.9958	0.9958
Pielou_J	0.8952	0.9115	0.8918	0.8919
Pd_faith	61.74	70.29	59.83	74.93

## Data Availability

The original contributions presented in this study are included in the article/Supplementary Material. Further inquiries can be directed to the corresponding authors.

## Supplementary Materials

The following supporting information can be downloaded at https://www.mdpi.com/article/10.3390/microorganisms13071653/s1, Text S1. The specific composition of BG11 medium; Figure S1. (a) Number of valid sequences of the four treatment groups, (b) Rarefaction curves, (c) Shannon-Wiener curves; Figure S2. (a) Venn diagrams of amplicon sequence variant (ASV) richness relationships and (b) principal coordinate analysis (PCoA) plot of the bacterial community structure among soil samples; Table S1. Interclass correlation of soil physicochemical property; Table S2. Mental test correlation; Table S3. The interaction between environmental factors and phylum; Table S4. The interaction between environmental factors and genus; Table S5. The top40 abundance of functional taxa based on PICRUSt2 Level3; Table S6. The top30 abundance of functional taxa based on FAPROTAX.

## References

[1] Ding B., Bai Y., Guo S., He Z., Wang B., Liu H., Zhai J., Cao H. (2023). Effect of irrigation water salinity on soil characteristics and microbial communities in cotton fields in Southern Xinjiang, China. Agronomy.

[2] Zhao X., Meng T., Jin S., Ren K., Cai Z., Cai B., Li S. (2023). The salinity survival strategy of *Chenopodium quinoa*: Investigating microbial community shifts and nitrogen cycling in saline soils. Microorganisms.

[3] Qadir M., Oster J.D., Schubert S., Noble A.D., Sahrawat K.L. (2007). Phytoremediation of sodic and saline-sodic soils. Adv. Agron..

[4] Gao L., Huang Y., Liu Y., Mohamed O.A.A., Fan X., Wang L., Li L., Ma J. (2022). Bacterial community structure and potential microbial coexistence mechanism associated with three halophytes adapting to the extremely hypersaline environment. Microorganisms.

[5] Chen H., Yu S., Yu Z., Ma M., Liu M., Pei H. (2024). Phycoremediation potential of salt-tolerant microalgal species: Motion, metabolic characteristics, and their application for saline–alkali soil improvement in eco-farms. Microorganisms.

[6] Tang L., Zhan L., Han Y., Wang Z., Dong L., Zhang Z. (2023). Microbial community assembly and functional profiles along the soil-root continuum of salt-tolerant *Suaeda glauca* and *Suaeda salsa*. Front. Plant Sci..

[7] Feng Y., Xu X., Liu J., Han J., Lu H. (2023). Planting *Suaeda salsa* improved the soil properties and bacterial community diversity in a coastal mudflat. Land Degrad. Dev..

[8] Ma C., Lei C., Zhu X., Ren C., Liu N., Liu Z., Du H., Tang T., Li R., Cui H. (2023). Saline-alkali land amendment and value development: Microalgal biofertilizer for efficient production of a halophytic crop-*Chenopodium quinoa*. Land Degrad. Dev..

[9] Shayesteh H., Jenkins S.N., Moheimani N.R., Bolan N., Bühlmann C.H., Gurung S.K., Vadiveloo A., Bahri P.A., Mickan B.S. (2023). Nitrogen dynamics and biological processes in soil amended with microalgae grown in abattoir digestate to recover nutrients. J. Environ. Manag..

[10] Zhang Z., Li T., Shao P., Sun J., Xu W., Zhao Y. (2023). Effects of short-term nitrogen addition on rhizosphere and bulk soil bacterial community structure of three halophytes in the Yellow River Delta. Land Degrad. Dev..

[11] Zhou T., Zhang L., Yang X., Wu Z., Yang Z., Wang J., Chen N., Ren X., Hu S. (2025). Prioritizing microbial functions over soil quality for enhanced multifunctionality in saline-sodic soil remediation. J. Environ. Manag..

[12] Wang J., Yasen M., Gong M., Zhou Q., Li M. (2024). Structural variability in the rhizosphere bacterial communities of three halophytes under different levels of salinity-alkalinity. Plant Soil.

[13] Lin H., Liu C., Li B., Dong Y. (2021). *Trifolium repens* L. regulated phytoremediation of heavy metal contaminated soil by promoting soil enzyme activities and beneficial rhizosphere associated microorganisms. J. Hazard. Mater..

[14] Peng M., Jiang Z., Xiang Z., Zhou A., Wang C., Wang Z., Zhou F. (2024). Genomic features of a plant growth-promoting endophytic *Enterobacter cancerogenus* JY65 dominant in microbiota of halophyte *Suaeda salsa*. Plant Soil..

[15] Li J., Hussain T., Feng X., Guo K., Chen H., Yang C., Liu X. (2019). Comparative study on the resistance of *Suaeda glauca* and *Suaeda salsa* to drought, salt, and alkali stresses. Ecol. Eng..

[16] Zhang Z., Wang L., Li T., Fu Z., Sun J., Hu R., Zhang Y. (2024). Effects of short-term nitrogen and phosphorus addition on soil bacterial community of different halophytes. mSphere.

[17] Ran J., Liu X., Hui X., Ma Q., Liu J. (2021). Differentiating bacterial community responses to long-term phosphorus fertilization in wheat bulk and rhizosphere soils on the Loess Plateau. Appl. Soil Ecol..

[18] Liu F., Mo X., Kong W., Song Y. (2020). Soil bacterial diversity, structure, and function of *Suaeda salsa* in rhizosphere and non-rhizosphere soils in various habitats in the Yellow River Delta, China. Sci. Total Environ..

[19] Zhang S., Pei L., Zhao Y., Shan J., Zheng X., Xu G., Sun Y., Wang F. (2023). Effects of microplastics and nitrogen deposition on soil multifunctionality, particularly C and N cycling. J. Hazard. Mater..

[20] Zhang Z., Sun J., Li T., Shao P., Ma J., Dong K. (2023). Effects of nitrogen and phosphorus imbalance input on rhizosphere and bulk soil bacterial community of *Suaeda salsa* in the Yellow River Delta. Front. Mar. Sci..

[21] Li Y., Lin Q., Wang S., Li X., Liu W., Luo C., Zhang Z., Zhu X., Jiang L., Li X. (2015). Soil bacterial community responses to warming and grazing in a Tibetan alpine meadow. FEMS Microbiol. Ecol..

[22] Wu Q., Chen Y., Dou X., Liao D., Li K., An C., Li G., Dong Z. (2024). Microbial fertilizers improve soil quality and crop yield in coastal saline soils by regulating soil bacterial and fungal community structure. Sci. Total Environ..

[23] Siddikee M.A., Zereen M.I., Li C., Dai C. (2016). Endophytic fungus *Phomopsis liquidambari* and different doses of N-fertilizer alter microbial community structure and function in rhizosphere of rice. Sci. Rep..

[24] Li X., Sardans J., Hou L., Gao D., Liu M., Peñuelas J. (2019). Dissimilatory nitrate/nitrite reduction processes in river sediments across climatic gradient: Influences of biogeochemical controls and climatic temperature regime. J. Geophys. Res. Biogeosci..

[25] Wang Y., Tang C., Wu J., Liu X., Xu J. (2013). Impact of organic matter addition on pH change of paddy soils. J. Soils Sediments.

[26] Gr S., Yadav R.K., Chatrath A., Gerard M., Tripathi K., Govindsamy V., Abraham G. (2021). Perspectives on the potential application of cyanobacteria in the alleviation of drought and salinity stress in crop plants. J. Appl. Phycol..

[27] Dineshkumar R., Kumaravel R., Gopalsamy J., Sikder M.N.A., Sampathkumar P. (2018). Microalgae as bio-fertilizers for rice growth and seed yield productivity. Waste Biomass Valorization.

[28] Mo X., Song Y., Chen F., You C., Li D., Liu F. (2023). Replacement of plant communities altered soil bacterial diversity and structure rather than the function in similar habitats of the Yellow River Delta, China. Ecol. Indic..

[29] Singh K. (2016). Microbial and enzyme activities of saline and sodic soils. Land Degrad. Dev..

[30] Alvarez A.L., Weyers S.L., Goemann H.M., Peyton B.M., Gardner R.D. (2021). Microalgae, soil and plants: A critical review of microalgae as renewable resources for agriculture. Algal Res..

[31] Zhang L., Li J., Bi H., Shi Q., Gong B. (2021). Effects of fulvic acid on tomato yield and rhizosphere soil microecology under different soil pH and phosphorus levels. China Veg..

[32] Li Y., Kang E., Song B., Wang J., Zhang X., Wang J., Li M., Yan L., Yan Z., Zhang K. (2021). Soil salinity and nutrients availability drive patterns in bacterial community and diversity along succession gradient in the Yellow River Delta. Estuarine, Coast. Shelf Sci..

[33] Chi Z., Zhu Y., Li H., Wu H., Yan B. (2021). Unraveling bacterial community structure and function and their links with natural salinity gradient in the Yellow River Delta. Sci. Total Environ..

[34] Guo W., Andersen M.N., Qi X., Li P., Li Z., Fan X., Zhou Y. (2017). Effects of reclaimed water irrigation and nitrogen fertilization on the chemical properties and microbial community of soil. J. Integr. Agric..

[35] Zhang Y., Zhang F., Abalos D., Luo Y., Hui D., Hungate B.A., García-Palacios P., Kuzyakov Y., Olesen J.E., Jørgensen U. (2022). Stimulation of ammonia oxidizer and denitrifier abundances by nitrogen loading: Poor predictability for increased soil N_2_O emission. Glob. Change Biol..

[36] Cai J., Fan J., Liu X., Sun K., Wang W., Zhang M., Li H., Xu H., Kong W., Yu F. (2021). Biochar-amended coastal wetland soil enhances growth of *Suaeda salsa* and alters rhizosphere soil nutrients and microbial communities. Sci. Total Environ..

[37] Xu L., Yu Q., Bai S., Wang M., Sun W., Xu S., Shi X., Lu J., Xie X., Qiu W. (2024). Soil organic carbon impact on soil physical properties through quantity and quality modifications. Soil Adv..

[38] Wang Z., Yang K., Yu J., Zhou D., Li Y., Guan B., Yu Y., Wang X., Ren Z., Wang W. (2022). Soil bacterial community structure in different micro-habitats on the tidal creek section in the Yellow River Estuary. Front. Ecol. Evol..

[39] Hao H., Yue Y., Chen Q., Yang Y., Kuai B., Wang Q., Xiao T., Chen H., Zhang J. (2024). Effects of an efficient straw decomposition system mediated by *Stropharia rugosoannulata* on soil properties and microbial communities in forestland. Sci. Total Environ..

[40] Qu Z., Li Y., Xu W., Chen W., Hu Y., Wang Z. (2023). Different genotypes regulate the microbial community structure in the soybean rhizosphere. J. Integr. Agric..

[41] Khalid M., Du B., Tan H., Liu X., Su L., Saeed ur R., Ali M., Liu C., Sun N., Hui N. (2021). Phosphorus elevation erodes ectomycorrhizal community diversity and induces divergence of saprophytic community composition between vegetation types. Sci. Total Environ..

[42] Xiao C., Guo S., Wang Q., Chi R. (2021). Enhanced reduction of lead bioavailability in phosphate mining wasteland soil by a phosphate-solubilizing strain of *Pseudomonas* sp., LA, coupled with ryegrass (*Lolium perenne* L.) and sonchus (*Sonchus oleraceus* L.). Environ. Pollut..

[43] Olanrewaju O.S., Babalola O.O. (2019). Streptomyces: Implications and interactions in plant growth promotion. Appl. Microbiol. Biotechnol..

[44] Kakumanu M.L., Ma L., Williams M.A. (2019). Drought-induced soil microbial amino acid and polysaccharide change and their implications for C-N cycles in a climate change world. Sci. Rep..

[45] Guo J., Chen Y., Lu P., Liu M., Sun P., Zhang Z. (2021). Roles of endophytic bacteria in *Suaeda salsa* grown in coastal wetlands: Plant growth characteristics and salt tolerance mechanisms. Environ. Pollut..

[46] Becker E.A., Seitzer P.M., Tritt A., Larsen D., Krusor M., Yao A.I., Wu D., Madern D., Eisen J.A., Darling A.E. (2014). Phylogenetically driven sequencing of extremely halophilic archaea reveals strategies for static and dynamic osmo-response. PLoS Genet..

[47] Tao J., Wang S., Liao T., Luo H. (2021). Evolutionary origin and ecological implication of a unique *nif* island in free-living *Bradyrhizobium* lineages. ISME J..

[48] An X., Wang Z., Jiao K., Teng X., Zhou R., Xu M., Lian B. (2023). Bacterial community characteristics in the rhizosphere of *Suaeda glauca* versus bulk soil in coastal silt soil modified by sea-sand and their implications. Front. Mar. Sci..

