# Supplementary material for: Irrigation of Suaeda salsa with Saline Wastewater and Microalgae: Improving Saline–Alkali Soil and Revealing the Composition and Function of Rhizosphere Bacteria
Source: Microorganisms. 2025 Jul 12;13(7):1653. doi: 10.3390/microorganisms13071653 (PMC12300775; doi:10.3390/microorganisms13071653)
Supplement: Supplementary file 1 [file microorganisms-13-01653-s001.zip › microorganisms-3714708-supplementary.pdf]

Supplementary Materials: Irrigation of Suaeda salsa with Saline Wastewater and Microalgae: Improving Saline–Alkali Soil and Revealing the Composition and Function of Rhizosphere Bacteria

Qiaoyun Yan [1], Yitong Zhang [1], Zhenting Xu [1], Wenying Qu [1,*], Junfeng Li [1], Wenhao Li [1,2,3], Chun Zhao [4,*] and Hongbo Ling [5]

[1] College of Water Conservancy and Architecture Engineering, Shihezi University, Shihezi 832000, China; 20222110020@stu.shzu.edu.cn (Q.Y.); 20221010007@stu.shzu.edu.cn (Y.Z.); 20221010026@stu.shzu.edu.cn (Z.X.); ljfshz@126.com (J.L.); lwh8510012@163.com (W.L.)
[2] Key Laboratory of Modern Water-Saving Irrigation of Xinjiang Production and Construction Group, Shihezi University, Shihezi 832000, China
[3] Key Laboratory of Northwest Oasis Water-Saving Agriculture, Ministry of Agriculture and Rural Affairs, Shihezi 832000, China
[4] Key Laboratory of the Three Gorges Reservoir Region's Eco-Environment, Ministry of Education, Chongqing University, Chongqing 400045, China
[5] Xinjiang Institute of Ecology and Geography, Chinese Academy of Sciences, Urumqi 830011, China; linghb@ms.xjb.ac.cn
* Correspondence: quwenying@shzu.edu.cn (W.Q.); pureson@163.com (C.Z.)

Table of contents

Text S1. The specific composition of BG11 medium.

Figure S1. (a) Number of valid sequences of the four treatment groups, (b) Rarefaction curves, (c) Shannon-Wiener curves.

Figure S2. (a) Venn diagrams of amplicon sequence variant (ASV) richness relationships and (b) principal coordinate analysis (PCoA) plot of the bacterial community structure among soil samples.

Table S1. Interclass correlation of soil physicochemical property.

Table S2. Mental test correlation.

Table S3. The interaction between environmental factors and phylum.

Table S4. The interaction between environmental factors and genus.

Table S5. The top40 abundance of functional taxa based on PICRUSt2 Level3.

Table S6. The top30 abundance of functional taxa based on FAPROTAX.

Text S1. The specific composition of BG11 medium.

The microalgae *Tetradesmus obliquus* ZYY1 was cultured in BG11 medium, which contained the following components: 1,500 mg/L $NaNO_3$, 40 mg/L $K_2HPO_4 \cdot 3H_2O$, 75 mg/L $MgSO_4 \cdot 7H_2O$, 36 mg/L $CaCl_2 \cdot 2H_2O$, 6 mg/L citric acid, 6 mg/L ferric ammonium citrate, 1 mg/L Na_2EDTA, 20 mg/L Na_2CO_3, 2.86 mg/L H_3BO_3, 1.81 mg/L $MnCl_2 \cdot H_2O$, 0.222 mg/L $ZnSO_4 \cdot 7H_2O$, 0.079 mg/L $CuSO_4 \cdot 5H_2O$, 0.39 mg/L $Na_2MoO_4 \cdot 2H_2O$, and 0.049 mg/L $Co(NO_3)_2 \cdot 6H_2O$. The chemicals used in this study were analytical reagent (AR) grade (> 99.0% purity) and purchased from Shanghai Macklin Biochemical Technology Co., Ltd. (Shanghai, China).

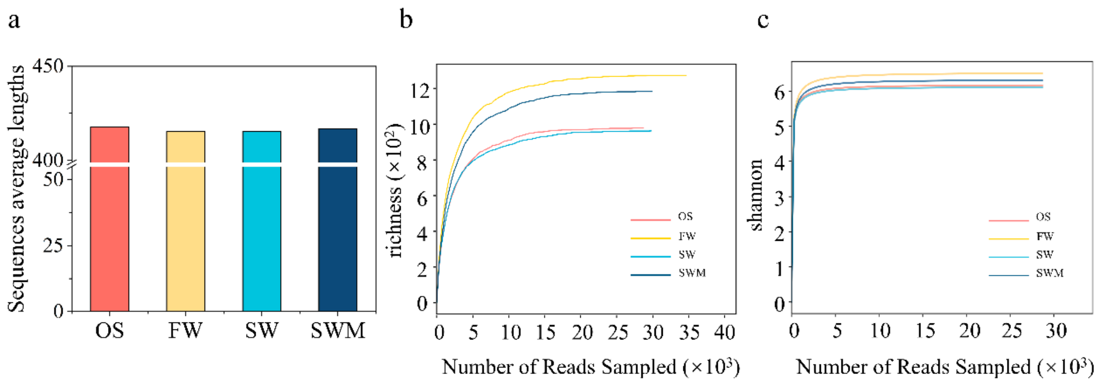

Figure S1. (a) Number of valid sequences of the four treatment groups, (b) Rarefaction curves, (c) Shannon-Wiener curves.

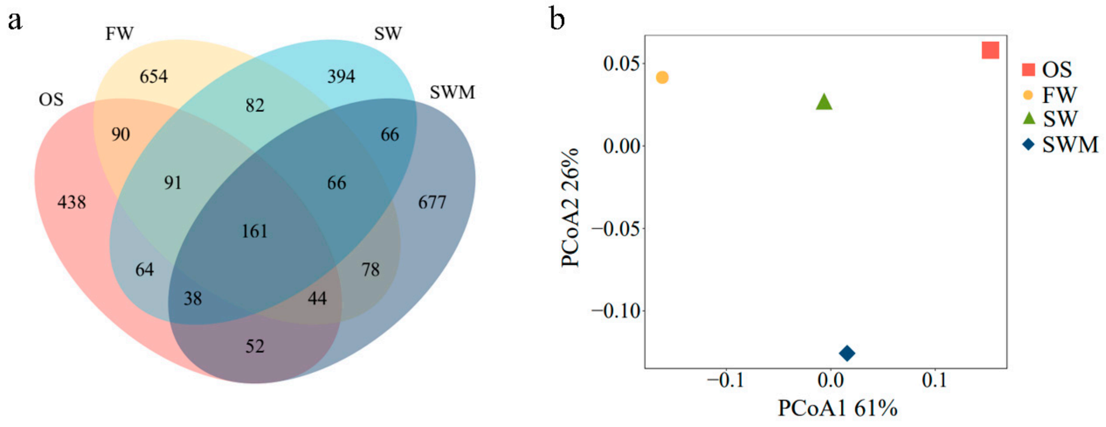

Figure S2. (a) Venn diagrams of amplicon sequence variant (ASV) richness relationships and (b) principal coordinate analysis (PCoA) plot of the bacterial community structure among soil samples.

Table S1. Interclass correlation of soil physicochemical property.

	pH	salt content	EC	TN	AN	AP	AK	SOC
pH	1.000							
salt content	-0.281	1.000						
EC	-0.313	0.963*	1.000					
TN	-0.486	-0.073	0.179	1.000				
AN	0.915	-0.139	-0.072	-0.147	1.000			
AP	0.413	-0.682	-0.481	0.515	0.572	1.000		
AK	-0.750	0.083	0.287	0.942	-0.454	0.218	1.000	
SOC	-0.421	-0.269	-0.019	0.980*	-0.123	0.628	0.896	1.000

* indicate that the correlation between the environmental factor is significant at the 0.05 level.

Table S2. Mental test correlation.

	pH	salt content	EC	TN	AN	AP	AK	SOC
Alpha diversity	0.009	-0.161	-0.151	-0.293	0.277	-0.157	-0.173	-0.272
Phylum	0.889*	-0.842	-0.670	0.227	0.636	-0.378	0.450	0.176
Genus	-0.126	-0.501	-0.497	0.916*	-0.408	-0.269	0.855	0.805*

* indicate that the correlation between the environmental factor is significant at the 0.05 level.

Table S3. The interaction between environmental factors and phylum.

	pH	salt content	EC	TN	AN	AP	AK	SOC
Acidobacteriota	-0.330	-0.813	-0.767	0.334	-0.432	0.393	0.347	0.488
Actinobacteriota	-0.970*	0.468	0.453	0.313	-0.914	-0.615	0.613	0.216
Bacteroidota	0.437	-0.467	-0.243	0.564	0.673	0.965*	0.254	0.631
Bdellovibrionota	0.284	-0.985*	-0.903	0.213	0.210	0.792	0.023	0.400
Chloroflexi	-0.628	-0.178	0.043	0.966*	-0.357	0.434	0.964*	0.970*
Firmicutes	-0.619	0.811	0.911	0.510	-0.325	-0.357	0.648	0.333
Gemmatimonadota	0.581	-0.366	-0.584	-0.901	0.204	-0.181	-0.917	-0.798
Myxococcota	0.711	-0.366	-0.562	-0.885	0.368	-0.077	-0.952*	-0.783
Patescibacteria	0.148	-0.842	-0.665	0.560	0.237	0.922	0.344	0.706
Proteobacteria	0.854	0.260	0.203	-0.543	0.839	0.035	-0.720	-0.582

* indicate that the correlation between the environmental factor is significant at the 0.05 level.

Table S4. The interaction between environmental factors and genus.

	pH	salt content	EC	TN	AN	AP	AK	SOC
Sphingomonas	0.907	0.069	-0.044	-0.728	0.775	0.001	-0.888	-0.722
Vibrionimonas	0.241	-0.830	-0.650	0.528	0.337	0.955*	0.286	0.672
Haliangium	0.721	-0.108	-0.319	-0.952*	0.408	-0.236	-0.998**	-0.900
Cellvibrio	0.117	0.197	0.440	0.706	0.506	0.576	0.505	0.637
Lysobacter	0.459	0.645	0.492	-0.716	0.375	-0.545	-0.693	-0.822
Nocardioides	-0.910	-0.090	0.012	0.697	-0.797	-0.020	0.865	0.697
Arthrobacter	-0.465	0.752	0.897	0.595	-0.115	-0.155	0.654	0.425
Mesorhizobium	-0.092	0.650	0.823	0.541	0.285	0.103	0.472	0.390
Bradyrhizobium	0.035	-0.808	-0.621	0.634	0.142	0.889	0.443	0.771
Rheinheimera	-0.145	0.842	0.948	0.338	0.173	-0.182	0.343	0.157
Streptomyces	-0.956*	0.475	0.441	0.249	-0.925	-0.661	0.559	0.153
Variovorax	0.262	-0.423	-0.174	0.709	0.538	0.943	0.431	0.763
Pontibacter	0.301	0.829	0.787	-0.312	0.412	-0.401	-0.319	-0.469
Bacillus	-0.106	0.782	0.584	-0.638	-0.236	-0.931	-0.421	-0.769
Burkholderia-Caballeronia-Paraburkholderia	0.233	0.625	0.408	-0.824	0.050	-0.782	-0.694	-0.920
Arenimonas	0.846	-0.091	-0.262	-0.877	0.597	-0.095	-0.986*	-0.833
Enterobacter	0.691	0.050	-0.165	-0.964*	0.405	-0.334	-0.990**	-0.943
Pseudomonas	0.406	-0.737	-0.546	0.489	0.539	0.997**	0.199	0.614
MND1	0.127	-0.097	-0.362	-0.886	-0.271	-0.597	-0.729	-0.832

Table S4 continued.

	pH	salt content	EC	TN	AN	AP	AK	SOC
Ellin6055	-0.906	-0.034	-0.070	0.254	-0.980*	-0.404	0.526	0.260
Rubrobacter	-0.760	0.802	0.854	0.416	-0.539	-0.528	0.627	0.246
Subgroup 10	0.894	-0.112	-0.261	-0.825	0.674	-0.010	-0.964*	-0.779
Gemmatimonas	-0.225	-0.033	-0.280	-0.689	-0.590	-0.707	-0.446	-0.652
Mycobacterium	-0.225	0.312	0.557	0.848	0.186	0.407	0.743	0.754
Altererythrobacter	-0.261	0.615	0.387	-0.659	-0.471	-0.984*	-0.386	-0.754
Hydrogenophaga	0.901	-0.040	-0.180	-0.802	0.708	-0.017	-0.947	-0.772
Rubellimicrobium	0.694	0.049	-0.165	-0.963*	0.408	-0.331	-0.991**	-0.942
Labrys	-0.116	0.800	0.923	0.380	0.218	-0.110	0.362	0.205
Blastococcus	-0.690	0.880	0.898	0.276	-0.500	-0.627	0.497	0.095
Nitrospira	0.819	-0.462	-0.615	-0.786	0.521	0.120	-0.919	-0.670

* and ** indicate that the correlation between the environmental factor is significant at the 0.05 and 0.01 level, respectively.

Table S5. The top40 abundance of functional taxa based on PICRUSt2 Level3.

Function	OS	FW	SW	SWM
ABC transporters	5.326	5.165	5.479	4.714
Ribosome	3.522	3.698	3.460	3.665
Purine metabolism	3.478	3.483	3.432	3.465
Two-component system	2.869	2.517	2.758	2.756
Oxidative phosphorylation	2.587	2.600	2.494	2.606
Pyrimidine metabolism	2.537	2.570	2.516	2.593
Arginine and proline metabolism	1.958	1.895	1.932	1.851
Pyruvate metabolism	1.858	1.857	1.884	1.847
Carbon fixation pathways in prokaryotes	1.844	1.884	1.804	1.913
Aminoacyl-tRNA biosynthesis	1.752	1.820	1.696	1.808
Glycine, serine and threonine metabolism	1.621	1.629	1.640	1.604
Glycolysis / Gluconeogenesis	1.538	1.622	1.622	1.579
Valine, leucine and isoleucine degradation	1.553	1.584	1.570	1.564
Butanoate metabolism	1.530	1.596	1.598	1.543
Amino sugar and nucleotide sugar metabolism	1.505	1.588	1.544	1.544
Propanoate metabolism	1.457	1.521	1.477	1.509
Citrate cycle (TCA cycle)	1.443	1.487	1.466	1.473
Alanine, aspartate and glutamate metabolism	1.429	1.448	1.425	1.432
Methane metabolism	1.341	1.378	1.388	1.360
Porphyrin and chlorophyll metabolism	1.332	1.339	1.340	1.351
Glyoxylate and dicarboxylate metabolism	1.324	1.323	1.362	1.326

Table S5 continued.

Function	OS	FW	SW	SWM
Homologous recombination	1.310	1.330	1.290	1.339
Phenylalanine, tyrosine and tryptophan biosynthesis	1.283	1.326	1.278	1.337
Valine, leucine and isoleucine biosynthesis	1.270	1.344	1.297	1.281
Bacterial secretion system	1.275	1.148	1.230	1.269
Cysteine and methionine metabolism	1.232	1.197	1.221	1.233
Peptidoglycan biosynthesis	1.137	1.164	1.110	1.140
Mismatch repair	1.116	1.109	1.092	1.145
Pentose phosphate pathway	1.065	1.111	1.109	1.097
Fatty acid degradation	1.042	1.132	1.092	1.096
Pantothenate and CoA biosynthesis	1.040	1.081	1.026	1.036
Protein export	1.018	1.047	1.005	1.032
RNA polymerase	0.980	1.037	0.976	1.002
Lysine biosynthesis	0.978	1.014	0.977	0.999
Fatty acid biosynthesis	1.018	0.970	0.968	0.995
Nitrogen metabolism	0.985	0.933	1.002	1.025
DNA replication	0.967	0.986	0.964	1.001
Flagellar assembly	1.079	0.870	0.970	0.931
Tryptophan metabolism	0.936	0.978	0.969	0.924
Histidine metabolism	0.936	0.966	0.942	0.952

Table S6. The top30 abundance of functional taxa based on FAPROTAX.

Function	OS	FW	SW	SWM
Chemoheterotrophy	28.83	31.94	35.13	29.89
aerobic_chemoheterotrophy	21.04	30.09	31.30	28.36
animal_parasites_or_symbionts	5.84	4.25	2.79	3.29
human_associated	5.84	4.18	2.79	3.29
human_pathogens_all	3.07	4.18	2.38	3.29
nitrate_reduction	3.86	2.34	2.57	2.94
human_pathogens_pneumonia	2.52	3.67	1.88	2.81
ureolysis	1.87	2.11	3.68	2.55
predatory_or_exoparasitic	3.46	1.82	1.11	1.49
fermentation	3.67	1.03	1.24	1.13
aromatic_compound_degradation	0.73	3.31	2.03	1.80
nitrogen_fixation	1.13	2.22	1.23	2.82
chitinolysis	2.32	1.10	2.45	0.42
human_gut	3.17	0.00	0.41	0.00
mammal_gut	3.17	0.00	0.41	0.00
nitrate_respiration	0.21	0.26	0.71	2.43
nitrogen_respiration	0.21	0.26	0.71	2.43
methylotrophy	1.63	0.26	0.76	0.48
methanol_oxidation	1.63	0.22	0.74	0.48
dark_hydrogen_oxidation	1.13	0.38	0.74	0.70
phototrophy	0.31	0.33	0.35	1.14

Table S6 continued.

Function	OS	FW	SW	SWM
nitrite_respiration	0.14	0.23	0.11	1.57
photoheterotrophy	0.27	0.33	0.27	1.10
chloroplasts	0.07	0.72	0.45	0.71
manganese_oxidation	0.38	0.73	0.31	0.25
nonphotosynthetic_cyanobacteria	0.03	1.10	0.13	0.39
hydrocarbon_degradation	0.78	0.04	0.30	0.03
intracellular_parasites	0.04	0.26	0.18	0.87
cellulolysis	0.10	0.53	0.21	0.54
aliphatic_non_methane_hydrocarbon_degradation	0.77	0.00	0.09	0.00
